# AI Education for Fourth-Year Medical Students: Two-Year Experience of a Web-Based, Self-Guided Curriculum and Mixed Methods Study

**DOI:** 10.2196/46500

**Published:** 2024-02-20

**Authors:** Areeba Abid, Avinash Murugan, Imon Banerjee, Saptarshi Purkayastha, Hari Trivedi, Judy Gichoya

**Affiliations:** 1 Emory University School of Medicine Atlanta, GA United States; 2 Yale New Haven Hospital New Haven, CT United States; 3 Mayo Clinic Phoenix, GA United States; 4 Indiana University-Purdue University Indianapolis, IN United States; 5 Department of Radiology Emory University Atlanta, GA United States

**Keywords:** medical education, machine learning, artificial intelligence, elective curriculum, medical student, student, students, elective, electives, curricula, curriculum, lesson plan, lesson plans, educators, educator, teacher, teachers, teaching, computer programming, programming, coding, programmer, programmers, self guided, self directed

## Abstract

**Background:**

Artificial intelligence (AI) and machine learning (ML) are poised to have a substantial impact in the health care space. While a plethora of web-based resources exist to teach programming skills and ML model development, there are few introductory curricula specifically tailored to medical students without a background in data science or programming. Programs that do exist are often restricted to a specific specialty.

**Objective:**

We hypothesized that a 1-month elective for fourth-year medical students, composed of high-quality existing web-based resources and a project-based structure, would empower students to learn about the impact of AI and ML in their chosen specialty and begin contributing to innovation in their field of interest. This study aims to evaluate the success of this elective in improving self-reported confidence scores in AI and ML. The authors also share our curriculum with other educators who may be interested in its adoption.

**Methods:**

This elective was offered in 2 tracks: technical (for students who were already competent programmers) and nontechnical (with no technical prerequisites, focusing on building a conceptual understanding of AI and ML). Students established a conceptual foundation of knowledge using curated web-based resources and relevant research papers, and were then tasked with completing 3 projects in their chosen specialty: a data set analysis, a literature review, and an AI project proposal. The project-based nature of the elective was designed to be self-guided and flexible to each student’s interest area and career goals. Students’ success was measured by self-reported confidence in AI and ML skills in pre and postsurveys. Qualitative feedback on students’ experiences was also collected.

**Results:**

This web-based, self-directed elective was offered on a pass-or-fail basis each month to fourth-year students at Emory University School of Medicine beginning in May 2021. As of June 2022, a total of 19 students had successfully completed the elective, representing a wide range of chosen specialties: diagnostic radiology (n=3), general surgery (n=1), internal medicine (n=5), neurology (n=2), obstetrics and gynecology (n=1), ophthalmology (n=1), orthopedic surgery (n=1), otolaryngology (n=2), pathology (n=2), and pediatrics (n=1). Students’ self-reported confidence scores for AI and ML rose by 66% after this 1-month elective. In qualitative surveys, students overwhelmingly reported enthusiasm and satisfaction with the course and commented that the self-direction and flexibility and the project-based design of the course were essential.

**Conclusions:**

Course participants were successful in diving deep into applications of AI in their widely-ranging specialties, produced substantial project deliverables, and generally reported satisfaction with their elective experience. The authors are hopeful that a brief, 1-month investment in AI and ML education during medical school will empower this next generation of physicians to pave the way for AI and ML innovation in health care.

## Introduction

Artificial intelligence (AI) and machine learning (ML) are poised to have a substantial impact in the health care space with many disruptive technologies on the horizon. Innovations in clinical care are increasingly impacted by the development and implementation of AI and ML, and as future clinicians, medical students need to become innovators and active participants in technological changes that will affect how they provide care for their patients. There is much excitement and curiosity among medical students about these technologies [1]. However, few programs exist to deliberately expose future physicians to their role in medicine, let alone to empower students to actively participate in AI and ML innovation [2]. While a plethora of high-quality web-based resources exist to teach programming skills and ML model development, there are few introductory curricula specifically tailored to medical students without a background in data science or programming. Additionally, there is little guidance provided to medical students on where to begin. Some medical societies do have AI outreach activities, but these are limited to trainees within their specialty [3-5].

The authors theorized that a 1-month elective for fourth-year students, composed of existing web-based resources and a project-based structure, would empower students to learn about the impact of AI and ML in their chosen specialty and begin contributing to innovation in their field of interest. The authors also aimed for the elective to be specialty-agnostic and customizable to each student’s career goals. The goal of this senior elective is to demystify AI and ML in health care, enabling students to have informed conversations about these technologies and participate in their clinical advancement. The target participant in the elective is any senior medical student with an interest in AI, with no prerequisites for technical, mathematical, or engineering skills.

In this paper, we evaluate the success of this elective over a 2-year period based on self-reported confidence scores in AI and ML. We also publish our curriculum for other educators who may be interested in its adoption.

## Methods

### Design

We built our elective following advice on designing medical electives with the principles articulated by Ramalho et al [6], which emphasize that a one-size-fits-all approach is often inadequate and that electives benefit from allowing students to carve their own paths. Creating a medical elective in an overloaded, overworked environment is nontrivial, but prior studies on peer-organized coursework gave us insights into the effectiveness of peer-organized research in building academic confidence, as well as the importance of clearly defined learning objectives [7,8].

### Technical and Nontechnical Tracks

Given the wide-ranging skill sets that medical students are equipped with before coming to medical school, this elective was offered in 2 tracks: Technical and Nontechnical. The Technical track was intended for the subset of students who were already competent computer programmers. This course did not aim to teach noncoding students how to code because it was expected that 1 month would not be sufficient time for students to make meaningful progress. Therefore, the Nontechnical track was offered to students with no technical background and focused on building a conceptual understanding of AI. Our goal for the Nontechnical track was to help students without a technical background develop a skill set and vocabulary that would enable them to participate in AI and ML evaluation and implementation processes in future collaborations with technical colleagues.

For both the Technical and Nontechnical tracks, the course was designed to address the following learning objectives:

1. Compare and contrast AI and ML.
2. State and differentiate various ML techniques (supervised/unsupervised, classification/regression, etc).
3. Appreciate the growing impact of ML in medicine, broadly and in the student’s chosen specialty.
4. Develop an intuition of how machines “learn.” Describe how neural networks are structured, trained, and evaluated. Learn vocabulary and concepts used to describe model training (loss functions, gradient descent, and backpropagation).
5. Understand the limitations and pitfalls of ML (reproducibility, interpretability, and bias).
6. Understand what kinds of medical problems can and cannot be solved by ML.
7. Describe issues that may arise in the implementation of an ML algorithm in clinical practice.
8. Discuss ethical issues that concern the use of ML in health care.

### Didactic and Project-Based Components

In this self-guided, web-based course, students were referred to existing web-based courses and relevant research papers to supplement these learning objectives (Multimedia Appendix 1 [9-22]) but were expected to guide their own learning beyond this. Students were asked to share and write down their personal goals at the beginning of the elective to guide their learning. They were also encouraged to spend time after each section on independent research to address lingering questions. The learning objectives and course resources were provided to students on a central document and students were able to follow along at their own pace. Because the course aimed to empower an individual student’s interests and career goals, the elective was designed to establish a baseline level of understanding for all students, while also allowing students the freedom to dive deeper into the areas they were drawn to. Students were supported by the course’s faculty advisor, a physician with substantial leadership and experience in AI and ML research.

### Project Deliverables

Students were then tasked with completing at least 1 of the following project-based deliverables, and encouraged to complete others as their interests dictated:

1. Complete a literature review on the state of AI and ML in the student’s chosen specialty.
2. Find and analyze 3 open-source health care data sets, considering strengths, weaknesses, and sources of error and bias.
3. Write a Project Proposal addressing a problem in the student’s chosen specialty that can be solved with AI, with a discussion surrounding the implementation complexities.
4. Technical track only: Train and evaluate a clinical ML algorithm.

Details on these projects are provided in Multimedia Appendix 2 [23].

The full curriculum is hosted on the Emory Health Care Innovations and Translational Informatics Lab GitHub repository [24].

This course was initially designed during the COVID-19 pandemic, and maintained a web-based format throughout the 2 years it has been offered. All recommended resources were freely available to students on the web, although some required institutional access. The students attended weekly web-based laboratory meetings to discuss their progress and to be exposed to more advanced research in AI and ML. Students were also encouraged to identify an additional advisor (beyond the elective director, who they met with once a week) within their chosen specialty, who could provide domain expertise for their projects.

### Qualitative Survey Data

Initially, the authors collected feedback from students qualitatively through one-on-one meetings; this feedback was used to improve the format and support structure of the elective. Beginning in October 2021, students were also asked for open-ended feedback on the strengths and weaknesses of the elective through anonymous surveys. They were asked:

- What was the most meaningful project or experience you completed during the elective? Do you intend to continue work on it past the end of the elective?
- Did you gain what you hoped to get out of this elective? Please explain.
- What resources were most useful to you during the elective?
- What could be most improved in the curriculum design of this elective?

### Quantitative Survey Data

Beginning in October 2021, quantitative pre and postelective surveys were implemented using Google Forms to assess the effectiveness of the elective format and resources provided. Students were asked to fill out formal surveys to rate their confidence in AI and ML concepts and in technical data science and coding skills.

Before starting the elective, students were asked:

- How familiar are you with AI or ML concepts? (Likert scale, 1-5)
- How would you rate your technical data science or coding experience? (Likert scale, 1-5)

After completing the elective, students were asked:

- Did you choose the Technical or Nontechnical Track?
- After completing this elective, how familiar are you with AI or ML concepts? (Likert scale, 1-5)
- After completing this elective, how would you rate your technical data science or coding experience? (Likert scale, 1-5)

### Statistical Analysis

Quantitative and discrete data from self-reported confidence scores was analyzed using the Wilcoxon rank sum test. Qualitative survey responses were reviewed in a descriptive manner rather than undergoing a formal analysis. Responses were manually examined for common themes, trends, and noteworthy insights, but no systematic coding framework was used and representative responses are included in the “Results” section.

### Ethical Considerations

This study was deemed exempt from review by Emory University’s institutional review board, under the category “Educational Tests, Surveys, Interviews, Observations.” This is justified based on anonymity and minimal risk to survey participants. All participants were able to opt out of this educational experience and from data collection. Survey data were collected anonymously. Students were not compensated for participation.

## Results

### Overview

This web-based, self-directed elective was offered on a pass-or-fail basis each month to fourth-year students at Emory University School of Medicine beginning in May 2021. A maximum of 3 students were allowed to enroll each month. As of June 2022, a total of 19 students had signed up and completed the elective. All students successfully met elective requirements and passed the course. The students represented a diverse range of chosen specialties: diagnostic radiology (n=3), general surgery (n=1), internal medicine (n=5), neurology (n=2), obstetrics and gynecology (n=1), ophthalmology (n=1), orthopedic surgery (n=1), otolaryngology (n=2), pathology (n=2), and pediatrics (n=1).

Given the limited time and open-ended nature of the course, students elected to spend varying amounts of time on each of the project components based on their interests and were not required to complete all 3 projects as long as they produced at least 1 significant deliverable. The vast majority of students (17 out of 19 students) chose the Nontechnical track. Most students (11/19, 58%) chose to focus their efforts on 2 of the 3 projects; 8 (42%) completed all 3 projects, and 1 (5%) submitted only a project proposal. Since the elective was intended to be flexible to students’ interests, students were evaluated on a pass-or-fail basis based on demonstrated effort as determined by the faculty advisor, rather than strict adherence to project deliverables. All students received a passing grade. Project proposals submitted by students were wide-ranging, including AI applications such as “Smartphone Detection of Anterior Uveitis,” “Predicting Postpartum Hemorrhage,” “Image Enhancement in Video Laryngoscopy,” and “Audiometry for Pediatric Heart Murmur Screening.” Four (25%) students indicated that they intended to continue working on their projects beyond the end of the elective.

### Qualitative Survey Results

Qualitative feedback collected from students before October 2021 (n=4) indicated that students wanted more support and guidance in their field of interest; given this feedback, the authors created more structure for the elective and encouraged students to find an additional specialty-specific mentor who could contribute domain expertise.

Students were asked if they gained what they hoped for from their elective experience. Students who sought a basic conceptual understanding reported satisfaction, but some reported an unmet desire for a deeper technical understanding:

- “I wanted to learn more generally how AI/ML can be used and is being used in medicine. I definitely achieved this goal.”
- “I feel that I learned AI/ML fundamentals, am now able to better read and understand AI/ML medical literature, and have thought through the essential design elements of an AI/ML proposal.”
- “I learned about the clinical applications of ML and how it is used to help rather than replace radiologists. I also have learned that the technology is advanced, but the application is still early in medicine.”
- “I found the course very valuable as an introduction to what ML is and how it is used. However, I had hoped to gain more insight into what research is being conducted in ML from a technical perspective and what these advances may mean from a translational perspective.”

Students were also asked what aspects of the course were most beneficial. Four students commented that the self-directed and flexible nature of the course was essential. Two students commented that the project proposal was the most essential element. Five (26%) students reported that they intended to continue working on their projects after the end of the elective month.

When asked for constructive feedback, 2 students commented that they desired more concrete guidance on the projects. Some students felt strained to finish the project proposal within 1 month, with one commenting that students should not expect to finish the proposal in 1 month, and 2 recommending future students pick a project as early as possible, rather than waiting until after the literature review and data set project.

### Quantitative Survey Results

After October 2021, students were asked to fill out formal surveys collecting feedback and self-reported confidence in skills gained during the elective. Fifteen students filled out the preintervention survey, and 12 students completed the postintervention survey. These results are shown in Table 1.

**Table 1:** Pre- and postintervention confidence scores in AI^a^ or ML^b^ concepts and technical skills.

		Mean (SD)		Median (IQR)
**“On a scale of 1-5, how well do you understand AI or ML concepts?”^c^**				
	Preintervention (n=15)	2.5 (1.3)	2 (3)	
	Postintervention (n=12)	4.1 (0.7)	4 (3)	
“**On a scale of 1-5, rate your technical data science skills”^d^**				
	Preintervention (n=15)	2.6 (1.4)	3 (0.25)	
	Postintervention (n=12)	1.9 (1.3)	1 (2)	

^a^AI: artificial intelligence.

^b^ML: machine learning.

^c^Relative difference is 66% and Wilcoxon rank sum *P* value is .003.

^d^Relative difference is –26% and Wilcoxon rank sum *P* value is .20.

## Discussion

### Principal Results

Students who participated in this elective were successful in diving deep into the potential of AI and ML in their area of interest and generally reported satisfaction with their elective experience. Students were asked to quantitatively rate their familiarity with both AI and ML concepts and coding or data science; the self-reported confidence scores for AI and ML rose by 66%, and these results were found to be statistically significant when analyzed by the Wilcoxon rank sum test. This exposure to AI and ML is a substantial improvement from the status quo, in which most medical students receive little to no exposure during the course of their training; in 1 study from 2022, 66.5% of students reported 0 hours of AI or ML teaching, and 43.4% had never heard the term “machine learning” [25]. Previous literature includes effective AI curricula developed for other types of health care trainees, such as radiology residents, but there is little to no literature on curricula evaluated for a fourth-year medical student audience as described in this paper [26,27].

Self-reported confidence in technical skills (coding and data science) fell by 26%, although this result was not found to be statistically significant. The authors attribute these results to an initial overconfidence prior to the elective, followed by an increased awareness of the technical complexity of model development after the elective.

Because this was a self-guided elective, student output varied with each student’s level of motivation and goals prior to entering the elective. Students who had defined a specific area of interest tended to benefit more from their experience than students who came in with no clear goals set. This course could be improved by providing further assistance early on in helping students to finalize a project area early so that they feel less strained by time toward the end of the month.

Students produced a wide range of deliverables in their chosen specialty. Since most fourth-year students have chosen their specialty and have established connections with faculty in their field, the self-guided nature of the course allowed flexibility for students to seek out appropriate mentors and propose reasonable projects in their areas of interest.

### Limitations and Future Directions

Limitations of this study include the small number of participants, especially in the Technical track, restricting the generalizability of this study. Only 2 (11%) students chose the Technical track, so there is insufficient data to evaluate this curriculum; this was likely due to the requirement that students interested in the Technical track have in-depth coding experience and receive approval from the course director to ensure a high likelihood of success. However, the authors recommend screening applicants to make sure that they do in fact possess the required level of comfort in coding before attempting to develop an ML model, as we observed a tendency for students to underestimate the complexity of this task. Based on qualitative observations that students spent more time than expected preparing data for training, the authors suggest providing select, cleaned data sets for students in the Technical track, allowing them to focus on model building, training, and testing.

Another substantial limitation is that assessments relied only on students’ self-reported confidence, which has been shown to be a flawed metric [28]. Further studies would benefit from a refined objective assessment tool of students’ competencies, as well as replication of this study at other medical schools.

Since launching this fourth-year elective, we have also adapted this curriculum to a shorter elective targeting second-year medical students and were invited to participate in a National Academies forum on AI for Health Profession Education to disseminate this curriculum to other learners [29].

### Conclusions

Overall, in the 2 years since launching the elective at Emory University School of Medicine, the authors have already seen substantial excitement and appreciation from senior medical students, with continued excitement in the elective’s third year. Most students entered the elective with minimal previous experience in AI and ML and were successful in completing self-guided research and proposing creative and realistic AI and ML projects. The authors are hopeful that a brief, 1-month investment in AI and ML education during medical school can lay the groundwork for these future physicians to continue to engage with AI and ML research and empower this next generation of physicians to pave the way for AI and ML innovation in health care.

## Appendix

Multimedia Appendix 1 Learning objectives and corresponding curated resources.

Multimedia Appendix 2 Project components and deliverables.

## Abbreviations

AI: artificial intelligence
ML: machine learning

## Data Availability

The data sets generated or analyzed during this study are not publicly available due to ensure participant confidentiality and privacy in compliance with the institutional review board exemption status, but are available from the corresponding author on reasonable request.
