# Supplementary material for: AI Education for Fourth-Year Medical Students: Two-Year Experience of a Web-Based, Self-Guided Curriculum and Mixed Methods Study
Source: JMIR Med Educ. 2024 Feb 20;10:e46500. doi: 10.2196/46500 (PMC10915728; doi:10.2196/46500)
Supplement: Multimedia Appendix 1 [file mededu_v10i1e46500_app1.docx]

# Multimedia Appendix 1

### Learning objectives and corresponding curated resources.

|  | Learning Objective | Curated Resources and Tasks |
| --- | --- | --- |
| 1 | Compare/contrast artificial intelligence and machine learning | - Complete Stanford’s “[Fundamentals of Machine Learning for Healthcare](https://www.coursera.org/learn/fundamental-machine-learning-healthcare)” course [9]; 12 h - Read Textbook, “Hands-On Machine Learning with Scikit-Learn, Keras, and TensorFlow, 3rd Edition”, chapter 1 [10]; 2 h |
| 2 | State and differentiate various machine learning techniques (supervised/unsupervised, classification/regression, etc) | - Covered in Stanford course and Textbook Ch1 above [9, 10] |
| 3 | Appreciate the growing impact of machine learning in medicine. | - Read “Machine Learning in Medicine” paper in *NEJM* [11]; 1 h - Read “A Short Guide for Medical Professionals in the Era of Artificial Intelligence” paper in *Nature* [12]; 1 h |
| 4 | Develop an intuition of how machines “learn”. Describe how neural networks are structured, trained, and evaluated. Learn vocabulary and concepts used to describe model training (loss functions, gradient descent, backpropagation). | - Watch 3-part video series on neural networks [13]; 2 h - Watch Emory MedAI workshop recording (optional) [14] - Train and test a model (without writing any code) using the Teachable Machine tool and a provided dataset [15] |
| 5 | Understand the limitations and pitfalls of ML (reproducibility, interpretability, bias). | - Read “Shortcuts: How Neural Networks Love to Cheat” article [16]; 1 h - Read “Artificial Intelligence versus Clinicians: Systematic Review of Design, Reporting Standards, and Claims of Deep Learning Studies” paper [17]; 1 h |
| 6 | Understand what kinds of medical problems can and cannot be solved by ML. | - Addressed in literature review, and through independent research (5 h) |
| 7 | Describe issues that may arise in implementation of a machine learning algorithm in clinical practice. | - Read “Developing a Delivery Science for Artificial Intelligence in Healthcare” paper [18]; 1 h |
| 8 | Discuss ethical issues that concern the use of ML in healthcare. | - Read “Bias in Predictive Algorithms” article [19]; 30 mins - Refer to “Can Machine Learning Solve Everything?” [video](https://www.coursera.org/learn/fundamental-machine-learning-healthcare/lecture/hbOBE/can-machine-learning-solve-everything) from Stanford course [9] - Read “AI recognition of patient race in medical imaging: a modelling study” paper [20]; 1 h - Read Implementing Machine Learning in Health Care — Addressing Ethical Challenges: paper [21]; 1 h - Read “Secure & Robust Machine Learning for Healthcare: A Survey” paper [22]; 1 h |
