# Supplementary material for: AI Education for Fourth-Year Medical Students: Two-Year Experience of a Web-Based, Self-Guided Curriculum and Mixed Methods Study
Source: JMIR Med Educ. 2024 Feb 20;10:e46500. doi: 10.2196/46500 (PMC10915728; doi:10.2196/46500)
Supplement: Multimedia Appendix 2 [file mededu_v10i1e46500_app2.docx]

# Multimedia Appendix 2

### Project components and deliverables

|  | Project | Steps / Deliverables | |
| --- | --- | --- | --- |
| 1 | Literature Review  (15 h) | - Read “How to Read Articles That Use Machine Learning: Users’ Guides to the Medical Literature” paper [23]; 1 h - Choose an area/specialty in which to conduct review: ___________ - Optional, but recommended: Identify a mentor in this field who can offer additional guidance: _______________ - Find and read 6-10 papers addressing the questions below.   - How can ML be used in this field?   - What types of ML are used?   - What kinds of datasets are used?   - What limitations exist? - What ethical considerations exist? - Possible research questions for Clinical AI project proposal - Briefly summarize learnings for each paper (2-3 bullets each). Turn in summaries. (1 h) | |
| 2 | Dataset Exercise | - Find ~3 open-source datasets for healthcare (such as those freely available on kaggle.com) - Prepare a written critique (1 page single-spaced) on the strengths and limitations of each dataset | |
| 3 | Clinical AI Project: ML Model or Conceptual Proposal | - Identify a problem and an AI-based solution: ____________ - Discuss with faculty mentor | |
|  |  | *If Technical Track:*  Train and evaluate a clinical ML algorithm in Python   - Refer to textbook for code samples and detailed technical guidance (remainder of text beyond Ch1) [10] - Find a dataset - Analyze and preprocess data - Build model and write code for training - Train and test model | *If Non-Technical Track:*  Draft an ML Project Proposal to address this question.  Address:   - Technical problem - Stakeholders - Data format and sources - Training and testing steps - Pilot and implementation plan |
